# Myoma expulsion after embolization: case report and literature review

**DOI:** 10.11604/pamj.2022.43.210.38030

**Published:** 2022-12-28

**Authors:** Maria Clara Amorim Silva, Rafael Everton Assunção Ribeiro da Costa, Wilson de Oliveira Sousa Júnior, Ariane Pereira Carvalho, Sabas Carlos Vieira

**Affiliations:** 1 Health Science Center, State University of Piauí, Teresina (PI), Brazil,; 2 Health Science Center, Federal University of Piauí, Teresina (PI), Brazil,; 3 Radiology, University Diagnostic Institute (UDI) 24-hour Clinic, Teresina (PI), Brazil,; 4 Tocogynecology, Oncocenter, Teresina (PI), Brazil

**Keywords:** Myoma, uterine artery embolization, postoperative complications, case report

## Abstract

Uterine artery embolization (UAE) is a very efficient treatment modality for myoma. A rare complication of this procedure is vaginal expulsion of the uterine myoma (expelled myoma) which may occur in 3 to 5% of cases during a period of 3 to 48 months. We report a case of myoma expulsion after embolization, discussing diagnosis and treatment. A literature review was also conducted. A 40-year-old patient sought medical care on 5/2/2021 with intermittent pelvic pain and hypermenorrhagia. Vaginal ultrasound revealed an enlarged uterus (253 cm^3^) with myomas. The largest intramural myoma measured 7 cm. Uterine artery embolization was performed on 11/11/2021, without any complications. On 12/7/2021, during clinical examination an expelled myoma was observed entirely inside the vaginal canal. A vaginal myomectomy was performed, without any complications. At 15 months after the initial follow-up, the patient is doing well.

## Introduction

Uterine artery embolization (UAE) is an effective treatment for symptomatic myomas since 1995 and symptom resolution occurs in 85-95% of cases [1,2]. Ideal candidates for the procedure are patients who wish to preserve their uterus, have no desire to conceive and those experiencing dysmenorrhea or hypermenorrhagia, in addition to premenopausal women [3]. As a result, ischemia, decreased myoma size occur, and endometrial perfusion is maintained. Finally, myoma remnants are absorbed by the preserved myometrium [1]. Nevertheless, a rare and late complication that merits discussion with candidates to UAE is vaginal expulsion of the uterine myoma (expelled myoma). The incidence of this complication is 3 to 5% of cases in a period of 3 to 48 months. It causes discomfort and requires surgical treatment. The process is due to uterine contractions secondary to the secretion of inflammatory prostaglandins, owing to the presence of necrotic material in the uterine cavity. Pedunculated submucosal myoma is the most common type of expelled myoma. Treatment of this complication requires antimicrobial coverage and the removal of all necrotic material by means of laparotomy, laparoscopy, hysteroscopy and/or hysterectomy to prevent sepsis [1].

The aim of this study is to report a case of myoma expulsion after embolization. The aspects of diagnosis and treatment were addressed and a literature review was also conducted.

## Patient and observation

**Patient information:** on 5/2/2021, a 40-year-old patient sought medical care, complaining of intermittent pelvic pain and hypermenorrhagia, that lasted up to 4 days. Systemic repercussions resulted in adynamia. The patient was a G0P0A0 woman and had no other comorbidities.

**Clinical findings:** on clinical examination, the uterus was palpable at about 4 cm from the symphysis pubis. Mucosae were pale +/4+ and no other alterations were observed.

**Figure 1:**
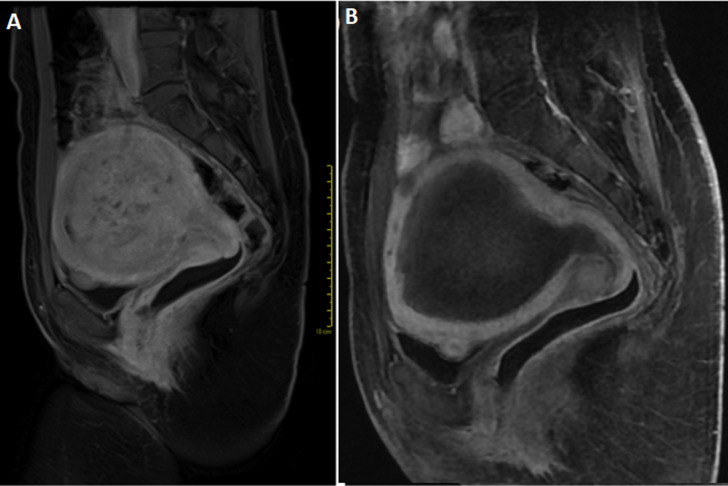
A) MRI before embolization (sagittal post contrast T1 fat saturation image demonstrated intense contrast enhancement of the myoma); B) MRI after embolization (sagittal post contrast T1 fat saturation image); it was observed that the myoma was no longer contrast-enhanced after embolization and its dimensions decreased

**Diagnostic assessment:** transvaginal ultrasonography (TVUS) demonstrated an enlarged uterus (253 cm^3^) with the presence of myomas. The largest myoma measured 7 cm (intramural location). Hemoglobin and hematocrit levels were 8.5 and 29.2, respectively. As a result of these findings, oral iron replacement and laparoscopic myomectomy were indicated. After these indications, the patient chose embolization, since she had no reproductive desire and wished to preserve the uterus. Magnetic resonance imaging (MRI) of the pelvis was performed (Figure 1A), revealing the presence of uterine myomas. The largest submucosal myoma measured 9 x 8.8 cm and there was also an intramural component. Total uterine volume was 597 cm^3^. Figure 1B shows pelvic MRI scan performed after the embolization procedure.

**Therapeutic interventions:** selective embolization of the uterine arteries was performed on 11/11/2021. Access was achieved via unilateral right femoral artery, using catheters and the injection of embolizing agents (microparticles of polyvynil alcohol). Uterine artery embolization procedure was performed under general anesthesia. The technique was successful and the patient was discharged from the hospital on the second postoperative day with minimal vaginal bleeding.

**Figure 2:**
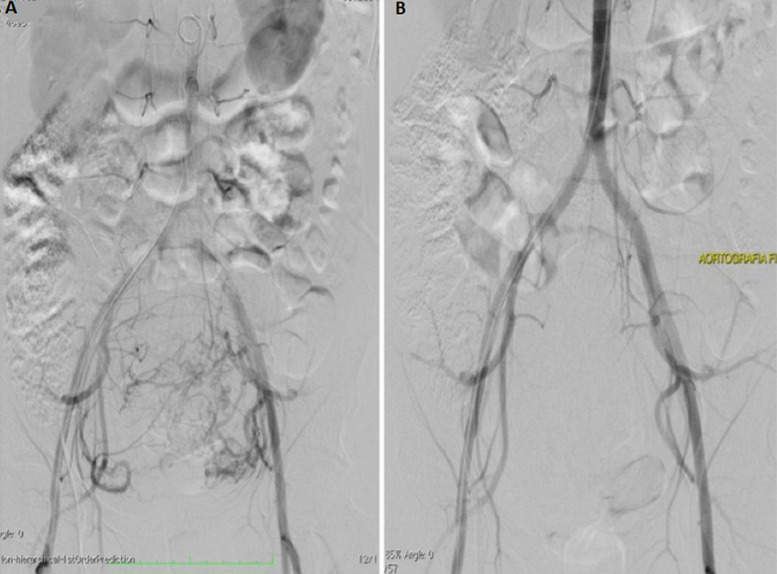
A) angiography before embolization showing uterine hypervascularization; B) angiography after embolization showing a devascularized myoma

**Figure 3:**
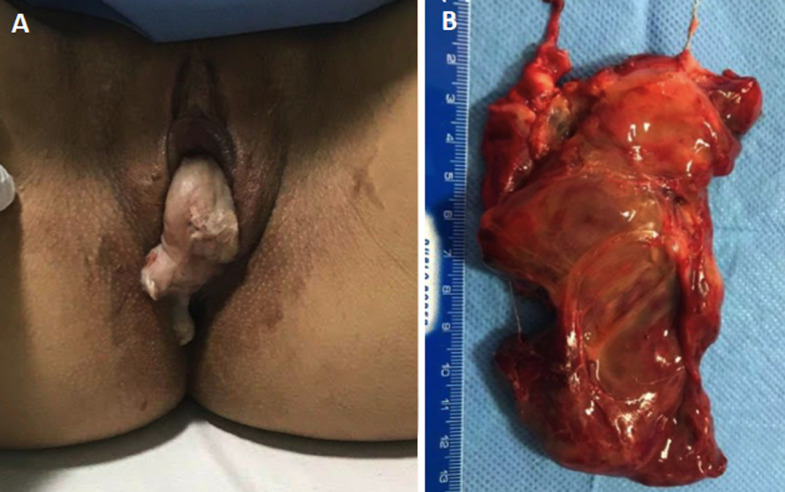
A) myoma externalized through the vagina; B) expelled myoma specimen

**Follow-up and outcome of interventions:** in the three following weeks, the patient sought the emergency department with complaints of pain. On the 25^th^ day after surgery, she began to feel vaginal discomfort with the sensation of mass externalization. Figure 2 shows the angiography performed before (Figure 2A) and after (Figure 2B) UAE. On 11/18/2021, the patient experienced intense pelvic abdominal pain, seeking urgent medical care. Abdominal computed tomography (CT) scan showed an image suggestive of hematometrium of around 240mL. She was medicated with analgesics with partial pain improvement. A new pelvic MRI was performed, revealing myoma devascularization. On 12/7/2021, she reported intense colicky pelvic pain and the sensation of a mass being eliminated through the vagina. On clinical exam, an expelled myoma was located entirely within the vaginal canal (Figure 3A). The patient was transported to the operating room, where vaginal myomectomy was performed under subarachnoid anesthesia without any complications. The surgical specimen was shown in Figure 3B. She was discharged from the hospital after 48 hours, maintaining treatment with iron replacement, tranexamic acid and ciprofloxacin for 7 days. On 12/18/2021, a levonorgestrel intrauterine device (IUD) was inserted into the uterus of the patient. Histopathology study of the surgical specimen confirmed that it was a necrotic leiomyoma. On 01/14/2022, since breakthrough bleeding persisted, an oral dienogest was combined for two months. At 15 months after the initial follow-up visit, the patient is currently asymptomatic and has a better quality of life.

**Diagnosis:** myoma expulsion after embolization.

**Informed consent:** this study was approved by the Institutional Review Board of the State University of Piauí, Teresina (PI), Brazil - reference number 5.535.238 (CAAE: 59331922.7.0000.5209). The patient signed the informed consent form (ICF).

## Discussion

Uterine leiomyomas are common benign tumors that usually occur in women over the age of 35. The most frequently reported symptoms are abnormal uterine bleeding (menometrorrhagia) and pelvic compression, occurring in about 20% of women [4,5]. UAE is a procedure in which the uterine arteries are embolized and the myoma is devascularized. Uterine size is also reduced. The technique has several advantages over hysterectomy (definitive treatment that prevents recurrences). UAE is a minimally invasive, well-tolerated, effective treatment modality that has lower complication rates and a shorter postoperative period [4,6]. It can also be performed in patients with absolute or relative contraindications to surgery, e.g. severe cardiopulmonary disease and thromboembolism, in addition to locally advanced pelvic cancers with active bleeding [6].

The majority of complications of UAE described in the literature are not life-threatening. Most patients experience only mild symptoms such as fever, pain and nausea [7]. Myoma expulsion is a rare complication. It can lead to endometritis and sepsis, which require rapid identification and treatment [5]. In the PubMed database, using the words myoma, embolization and expulsion, we found only 18 cases reported in the literature (Table 1) [2,3,5,7-14]. Initial treatment included intravenous fluids, antibiotics and curettage of necrotic remnants within the endometrial cavity. In the lack of response, hysterectomy is required to prevent patient risk [5]. When this diagnosis is suspected, MRI has significant value due to its higher sensitivity. It is also the imaging modality of choice before and after UAE, since it identifies the size, position and number of myomas in the uterine cavity [6].

**Table 1:** case studies described in the literature of myoma expulsion following UAE, according to author, year, number of cases, age (mean, in years), reason for UAE, post-UAE complications and procedures

Author	Year	Number of cases	Age (mean, in years)	Reason for UAE	Complications (post-UAE)	Procedures (post-UAE)
Martins *et al*.	2016	1	44	Acute bleeding	4 days: a 15-cm protruding necrotic mass through the vagina; uterine necrosis and endomiometritis	Suction curettage; hysterectomy
Marret *et al*.	2004	1	49	Menometrorrhagia	4 years: expelled a necrotic mass from the cervix to the vagina	Removed and the patient was asymptomatic 6 months after the procedure
do Amaral *et al*.	2021	1	36	Menometrorrhagia (10 years)	15 days: expulsion of the mass	Dilatation and curettage - remove exsudate and residual myoma
Felemban *et al*.	2001	1	43	Menorrhagia (1 year)	21 days: expulsion of solid fibroid tissue through the vagina (2.6x2.1x1.1 cm); 27 days: tissue expulsion (5.0x3.0x1.5 cm); 35 days: tissue expulsion (9.0x5.0x3.0 cm) - 3 myomas together	Asymptomatic repeated expulsion; transvaginal ultrasound 1 month after - did not reveal myoma
Hehenkamp *et al*.	2004	1	54	Menorrhagia	73 days: myoma protruding into the vagina	Surgery with general anesthesia - painful manipulation (myoma connected to the interior of the uterus); 4 weeks after the surgery: MRI showed a reduced uterus without leiomyomas (214 cm^3^)
Redecha Jr *et al*.	2009	1	32	Menometrorrhagia (2 years)	21 days: tip of necrotic myoma protruding from the cervix	Myoma ablation retained in the uterine cavity under general anesthesia; 1 week after the procedure: US revealed the absence of myomas and a normal uterine size
Park *et al*.	2005	8 (6.5%)	40.3	Menorrhagia	70.5 days: spontaneous expulsion (7 cases)	1 case: hysteroscopy. Resolution of all symptoms after the passage of the myoma.
Pollard *et al*.	2001	1	40	Menorrhagia	14 days: protruding cervical myoma	Hysterectomy
Laverge *et al*.	2003	1	45	Menorrhagia (6 months)	28 days: protruding necrotic mass through the vagina (150x140x30 mm); 29 days: second protruding mass (110x100x60 mm); 49 days: third protruding mass	Mass was removed and sent to the histology laboratory; 1 year after hysteroscopy: transvaginal ultrasound revealed a normal uterine size; complete resolution of symptoms
Kroencke *et al*.	2003	1	48	Menorrhagia	210 days: vaginal discharge with fragments	Magnetic resonance imaging (MRI) after UAE showed regular uterine architecture
Abbara *et al*.	1999	1	48	Menorrhagia	18 days: protruding mass through the vagina (6.7x5.5x4.5 cm)	Removed two days after a routine visit

During myoma expulsion, which may consist of prolonged periods of desquamation, patients may experience symptoms ranging from minimal discomfort to vaginal discharge, bleeding and low fever. In a study by Kroencke *et al*. data was validated according to a patient report [13]. She described that desquamation was part of the expulsion process and occurred for 7 months after UAE [7]. Regarding contraindications, myomas larger than 10 cm and submucosal myomas do not invalidate the performance of the procedure. Although there is a higher association between complications and larger myomas, complications most likely arise from the location rather than the size of the myoma [5]. In contrast, a successful procedure is defined as improvement in symptoms and no need for further treatment. In addition, the reduction in uterine size varies from 40 to 70% after undergoing the procedure [7].

The effects of UAE on women suffering from infertility and abortions are still not fully known. Ravina *et al*. conducted a study showing unplanned pregnancy in twelve women after undergoing the technique, with 58.3% occurring in women over 37 years of age. This study proved that conception was facilitated by decreasing the amount of myomas and restoring normal uterine configuration [2,7,15].

In this study, the case report described a 40-year-old woman, within the mean age (43.5 years) calculated in the 11 case studies published in the literature, as shown in Table 1. In this case, it is worth mentioning that after the procedure the only minor complication was pain, which is considered a common symptom. In the presence of a protruding mass, the procedure of choice was hysteroscopy for removal of necrotic material. The patient had a good response to hormone treatment, with resolution of menstrual bleeding.

## Conclusion

Myoma expulsion after UAE has not been commonly reported in the literature. To the best of our knowledge, only 18 cases have been described in the literature to date. Most of these patients were treated with analgesics and antibiotics, in addition to the removal of necrotic remnants by means of a simple procedure - hysteroscopy with curettage. Nevertheless, in severe cases due to failure of myometrial perfusion, hysterectomy is the treatment of choice.
